# Should we measure the central venous pressure to guide fluid management? Ten answers to 10 questions

**DOI:** 10.1186/s13054-018-1959-3

**Published:** 2018-02-23

**Authors:** Daniel De Backer, Jean-Louis Vincent

**Affiliations:** 1 Department of Intensive Care, CHIREC Hospitals, Université Libre de Bruxelles, Boulevard du Triomphe 201, B-1160 Brussels, Belgium; 2 Department of Intensive Care, Erasme University Hospital, Université Libre de Bruxelles, Brussels, Belgium

**Keywords:** Central venous pressure, Hemodynamics, Fluid responsiveness, Cardiac output

## Abstract

The central venous pressure (CVP) is the most frequently used variable to guide fluid resuscitation in critically ill patients, although its use has been challenged. In this viewpoint, we use a question and answer format to highlight the potential advantages and limitations of using CVP measurements to guide fluid resuscitation.

The central venous pressure (CVP) remains the most frequently used variable to guide fluid resuscitation in critically ill patients [1]. Use of the CVP has been challenged in many studies, which have reported that other indices are better than the CVP for predicting the response to intravenous fluids [2, 3]. However, we would argue that rather than simply banning the use of CVP because its predictive value may be less than desired in some situations, it is more useful to clearly understand the potential advantages and limitations of CVP measurements in order to improve its use. Moreover, the value of CVP measurements for guiding fluid resuscitation may go well beyond their simple use as a predictor of fluid responsiveness. In this viewpoint, we discuss the pros and cons of CVP for guiding fluid resuscitation (Table 1).

**Table 1:** The pros and cons of central venous pressure (CVP) for fluid management

	Pro	Con
Measurements	Easy to measure	Errors in measurements
	Minimal apparatus	Influence of mechanical ventilation
	Cheap	Influence of abdominal pressure
CVP for fluid responsiveness	The predictive value of extreme CVP values (CVP < 6–8 mmHg and CVP > 12–15 mmHg) is satisfactory [7, 8]	The predictive value for fluid responsiveness is lower with CVP than with dynamic indices
CVP as a safety value	During a fluid challenge, a given CVP value can be used as a safety value	This safety value should be individually determined as there is no predefined safe upper level of CVP
CVP as a target value	In circulatory failure, this population-based approach may be used to ensure that the majority of the patients achieve a satisfactory hemodynamic goal	In circulatory failure, a significant number of patients may be submitted to excessive fluid administration whereas other patients may require additional fluid administration
		In patients without indices of hypoperfusion, this approach is not recommended as it could lead to unnecessary fluid administration [19]
Influence of mechanical ventilation	The CVP represents the back pressure of all extrathoracic organs	The CVP may fail to reflect intravascular pressure during mechanical ventilation
CVP can be used to evaluate the response to fluids	An increase in CVP indicates an increase in preload	The increase in CVP indicates the increase in preload but does not indicate the response to fluids; in fluid responders the increase in CVP should be minimal (with a large increase in cardiac output) while in nonresponders the increase in CVP is larger
	An absence of change in CVP during fluid administration indicates that insufficient fluids were administered to manipulate preload	

## Question 1: Are the factors that influence CVP too numerous to make it meaningful?

We agree that the CVP is influenced by many factors, but this is also true for other variables such as blood pressure, heart rate, and cardiac output—does that mean we should not measure them? No, rather we need to understand which factors influence these measurements and how, in order to use them optimally.

CVP is an indicator of right ventricular and, to a lesser extent, left ventricular preload. CVP also reflects the limit to venous return and informs about right ventricular function. As such, CVP measurements may be helpful to guide fluid management. However, CVP is also affected by thoracic, pericardial, and abdominal pressures, which makes its interpretation more complicated. Indeed, although the CVP measured in these conditions overestimates the transmural CVP and may thus fail to reflect the true loading conditions of the right ventricle, it does represent the limit to venous return and the back pressure of all extrathoracic organs. In particular, the risk of peripheral edema, ascites, renal, and liver impairment is related to the absolute CVP value [4, 5].

## Question 2: Can a given CVP value determine whether a patient is fluid responsive?

**Fig. 1:**
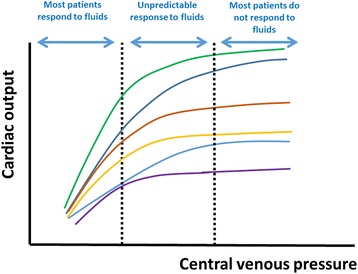
Frank-Starling relationship in individual patients. At low central venous pressure (CVP) values, most patients respond to fluids. At high CVP values, most patients do not respond to fluids. Between the two dotted lines, the response to fluids cannot be predicted from the CVP

According to the Frank-Starling relationship, stroke volume increases with CVP until a plateau is reached (Fig. 1). It may thus sound attractive to try to reach a CVP value that is close to the plateau. Unfortunately, this approach has limited value, because there is wide inter-patient variability in the slopes. Accordingly, it is very difficult to predict the response to fluids based on a single CVP value. In a trial investigating 150 fluid challenges performed in 96 septic patients being treated with mechanical ventilation, Osman et al. observed that baseline CVP was similar in responders and nonresponders so that the predictive value of CVP was low [6].

The classical statistical approach to evaluating the predictive value of a test is often limited as it considers the risk of error independent of the clinical situation. Nevertheless, it is less risky to administer fluids in a nonresponsive patient with a low CVP than one with a high CVP since the risk of edema formation is obviously lower.

The presence of an “extreme” CVP value may be more helpful to guide fluid administration than intermediate values. In a recent systematic review by Eskesen et al., including 1148 patients from 51 studies that evaluated the response to a fluid bolus and reported CVP, the overall predictive value of CVP was poor [7]. However, approximatively two thirds of the patients with a CVP less than 8 mmHg but only one third of patients with CVP values greater than 12 mmHg responded to fluids. In another study [8] that included 556 patients (460 of whom were from eight studies published by the authors of the review, and interestingly three of these studies were not included in the other systematic review [7]), the authors used the gray zone approach to determine CVP values between which no decisions on fluid responsiveness could be taken. A positive response to fluids was observed when CVP values were less than 6 mmHg but was unlikely when values were greater than 15 mmHg [8].

These data suggest that extreme CVP values can help to guide the response to fluids whereas intermediate values cannot (Fig. 1). It may thus be wise to refrain from administering fluids when the CVP is markedly elevated.

## Question 3: Can changes in CVP during fluid administration be used as an indication of the response to fluids?

Yes and no! The increase in CVP does not reflect changes in cardiac output! According to Guyton, venous return is inversely related to the difference between mean systemic pressure (Pms) and CVP, suggesting that the higher the CVP, the lower the cardiac output. The role of the heart in this context is to try to keep CVP as low as possible to maintain the gradient between Pms and CVP. The goal of fluid resuscitation is to increase the gradient between Pms and CVP by increasing Pms, not by increasing CVP itself. Analyzing data published by Cecconi et al. [9] who measured cardiac output, CVP, and Pms during fluid challenge in postoperative patients, it becomes clear that Pms increased similarly in fluid responders and nonresponders. In contrast, CVP increased minimally in responders so that the gradient for venous return and hence cardiac output increased. In the nonresponders, CVP increased markedly so that the gradient for venous return and thus cardiac output remained unchanged. Hence, cardiac tolerance to volume loading is a greater determinant than changes in Pms for determining the response to fluid challenge.

**Fig. 2:**
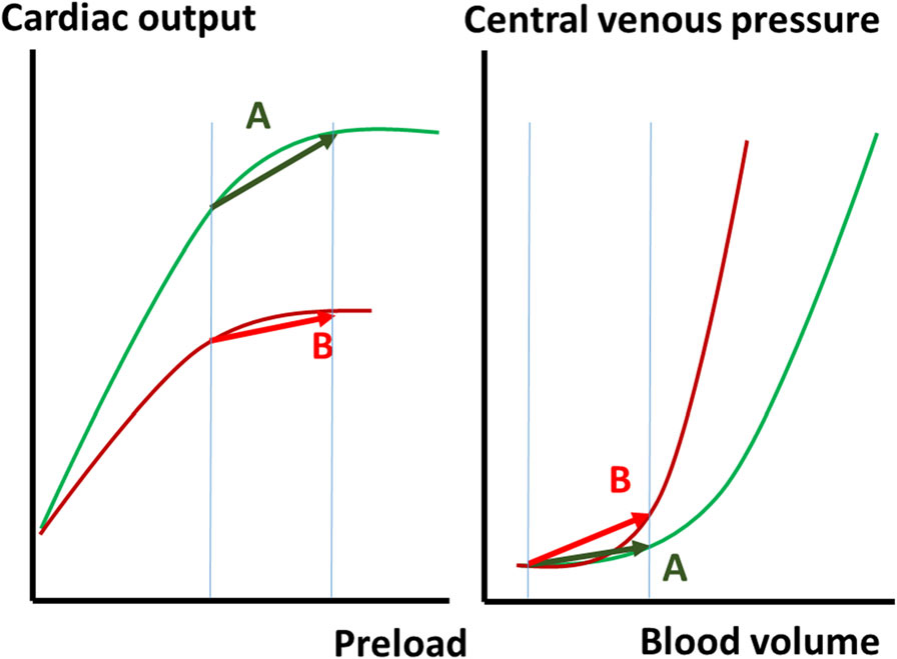
Relationship between preload, cardiac output, and central venous pressure (CVP). Relationship between cardiac output and preload (left panel) and between CVP and blood volume (right panel) in a fluid responder (**a**) and a nonresponder (**b**). In the fluid responder, the administration of fluids increases blood volume and cardiac preload; the increase in preload is associated with a large increase in cardiac output and a minimal increase in CVP. In the fluid nonresponder, the same increase in blood volume and preload is associated with no change in cardiac output and major changes in CVP. Accordingly, an increase in CVP cannot be used to suggest a positive response to fluids. Volume measurements better evaluate changes in preload in preload-responsive patients while pressure measurements better evaluate changes in preload in preload-nonresponsive patients

Accordingly, changes in CVP during fluid challenge should be analyzed together with changes in cardiac output; a large increase in CVP with minimal change in cardiac output indicates poor tolerance to fluids, whereas minimal change in CVP together with an increase in cardiac output indicates fluid responsiveness (Fig. 2).

Considering changes in CVP without taking into account changes in cardiac output can be very misleading as one may erroneously consider that the greater the change in CVP the larger the increase in preload and thus potentially the greater the effect of fluids. However, the opposite effect may be true, i.e., cardiac tolerance may be poor and the increase in cardiac output may have been minimal. Without cardiac output measurements, the only conclusion that can be drawn from the increase in CVP is that preload effectively increased during fluid administration, but the patient’s response to fluids is unknown.

Changes in CVP during a fluid bolus can be used to predict the response to further fluid administration. In 80 surgical patients, Hahn et al. observed in nonresponders that fluid response to a second fluid challenge was more likely when changes in CVP during the first fluid administration were minimal (the odds ratio for later becoming a fluid responder was only 0.11 (95% confidence interval, 0.03–0.38) if CVP increased, compared to when CVP did not increase) [10].

Hence, if anything, changes in CVP should be minimal, but accompanied by an increase in cardiac output; otherwise it implies that preload was not affected.

## Question 4: Can CVP be used as a safety variable?

One way to look at changes in CVP during a fluid challenge is to consider CVP as a safety variable. After all, the decision to administer fluids is based on a benefit/risk analysis; the benefits are related to an increase in cardiac output, and the risks to an increase in hydrostatic pressure increasing edema formation. CVP is an excellent variable to estimate the risk associated with extrathoracic organ congestion. Limiting CVP in liver surgery is associated with less risk of bleeding and better perioperative outcomes [11]. Similarly, the incidence of acute kidney injury is increased in patients with sepsis or with congestive heart failure who have elevated CVP values [4, 12]. In patients with sepsis, the risk of developing acute kidney injury increased with the mean CVP values over the first 12 h after admission [4]. In patients with congestive heart failure, those in the two upper quartiles of CVP had more severe renal impairment compared to the other quartiles, even though cardiac output was similar in the four quartiles [12]. These data strongly demonstrate an association between an elevated CVP and an increased risk of developing acute kidney injury, but they do not demonstrate a causal link.

Importantly, the CVP may fail to reflect the risk of developing pulmonary edema, which depends on capillary pressure and hence on left atrial pressure. In these conditions, measurements of pulmonary artery occluded pressure may be preferred as safety variables for the lungs, and/or measurements of extravascular lung water to estimate the severity of lung edema.

## Question 5: Is there a safe CVP value?

This is a difficult question, as no clear cut-off value can be identified. As mentioned earlier, an elevated CVP may perhaps be more a marker of severity of cardiovascular and/or respiratory function than a factor contributing to a poor outcome; thus, it would be illogical to prevent it from reaching high values. In a study by Boyd et al. [13] in patients with septic shock, a CVP less than 8 mmHg at 12 h was associated with improved survival, but it was no longer associated with outcome at days 1–4. As different cut-off values for CVP have been reported in different studies, and as a given CVP value may or may not be associated with a worse outcome, it seems logical to determine the upper limit of CVP on an individual basis, weighing the potential benefit/risk of further fluid administration against the potential benefit/risk of alternative interventions. This individually determined upper limit can be used as a value at which further fluid administration should be restricted. Nevertheless, one should try to keep CVP as low as possible as long it remains associated with adequate tissue perfusion.

## Question 6: Should fluids be administered to reach predefined CVP values?

This concept is the basis of some resuscitation algorithms including that used by Rivers et al. [14] in their study on early goal-directed therapy and the large subsequent trials evaluating this strategy [14–17]. Of note, the same CVP targets were used in the goal-directed and control arms so that no conclusions can be made on the effectiveness of this approach based on these trials. In the Protocolized Care for Early Septic Shock (ProCESS) trial [15], CVP measurements were not mandated in the usual care arm. No difference in survival or organ dysfunction was reported between the usual care arm and the two other arms, but it should be noted that a central line was inserted in the majority of patients in the usual care arm and we do not know whether a CVP value was targeted in these patients.

Physiologically, it does not seem reasonable to target a specific CVP value because the CVP value above which a patient would not respond to fluids is highly variable. As indicated earlier, each patient follows their own Frank-Starling curve, and only extreme CVP values carry some predictive value for fluid responsiveness. Hence, targeting a specific CVP value is only valid at the population level. Applying bootstrap analysis on data obtained from 564 critically ill patients submitted to a fluid challenge, Biais et al. [8] nicely demonstrated that the likelihood of responding to fluid decreased progressively with increasing CVP values and that almost no patient responded to fluids when presenting CVP values greater than 20–22 mmHg. Nevertheless, it would be dangerous to target such high values of CVP as many patients would be exposed to the detrimental effects of fluids while still being nonresponsive. Target CVP values of 8–12 mmHg became almost “standard” after the study by Rivers et al., who determined them a priori [14]. These values represent a reasonable target as the majority of patients respond to fluids when CVP is less than 8 mmHg and only a minority when it is greater than 12 mmHg [7, 8]. Nevertheless, using these values of CVP to guide fluid administration is far from perfect and should only be applied when more accurate predictors of fluid responsiveness cannot be obtained.

In stabilized patients, it is clear that no attempt should be made to increase CVP to specific target values. In the FACT trial, patients with acute respiratory distress syndrome (ARDS) [18] were randomized after initial resuscitation to liberal or conservative fluid therapy, targeting higher or lower values of CVP (or pulmonary artery occlusion pressure (PAOP)). There were no differences in survival between the groups, but patients in the conservative strategy group were more rapidly weaned from mechanical ventilation. In a recent exploratory analysis of this trial, the risk of death was increased in the liberal group compared to the conservative group when basal CVP was between 0 and 10 mmHg but not at higher values of CVP [19], suggesting a harmful effect of fluid administration to target a given CVP value when this was not needed.

## Question 7: Are CVP measurements reliable enough? Are there not too many technical problems?

The reliability of CVP measurements has been questioned, with errors related both to positioning of the zero level as well as reading errors. Although these potential errors should be acknowledged as a clear limitation, they are not restricted to CVP measurements and, more importantly, adequate training should limit the risk of such errors occurring.

## Question 8: Are measurements of end-diastolic volumes not preferable (more physiologic) as intraventricular volumes better reflect preload than pressures?

**Fig. 3:**
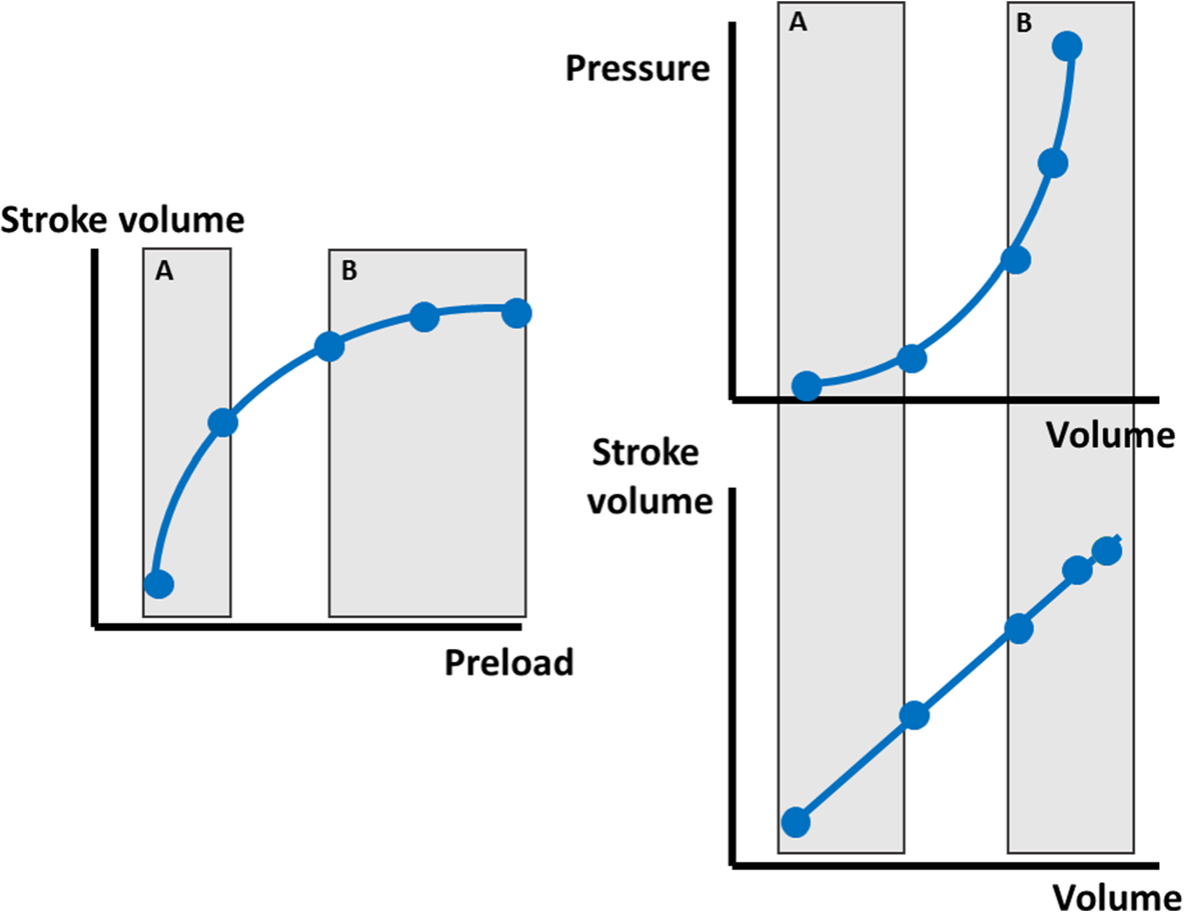
Relationship between preload, end-diastolic volumes, and pressures. Volume measurements better evaluate changes in preload in preload-responsive patients (**a**) while pressure measurements better evaluate changes in preload in preload-nonresponsive patients (**b**)

Indeed, measurements of end-diastolic volume by transpulmonary thermodilution or echocardiography reflect cardiac preload better than do intravascular pressures, including CVP. In healthy volunteers, changes in stroke volume during fluid loading correlated better with changes in cardiac volumes than with CVP [20]. Of note, volumes better predict fluid responsiveness on the steep part of the Starling relationship, whereas on the plateau pressures indicate better that the patient has reached the limits of filling (Fig. 3). In addition, measurements of intravascular pressures are more physiologic in terms of the Starling relationship of the vessels; indeed, edema formation depends on intravascular pressures and not on volumes.

In fact, this question is actually a bit ‘passé’ as there is no evidence that volumes are better than pressures for fluid management in critically ill patients. The only exception is probably the abdominal compartmental syndrome, in which CVP is markedly increased due to the increase in intrathoracic pressure, while cardiac volumes are markedly reduced.

## Question 9: Are dynamic indices of fluid responsiveness better for guiding fluid administration?

Dynamic indices of fluid responsiveness, such as pulse pressure variation (PPV) or stroke volume variation (SVV), or changes in cardiac output during a passive leg raise, reflect that the heart is on the ascending part of the Starling relationship. Given their excellent predictive capacities for fluid responsiveness, these tests are now included in recent guidelines [21, 22]. Unfortunately, PPV or SVV can only be used reliably in a minority of the patients, who are mechanically ventilated, sedated and without arrhythmias. The passive leg raising test has fewer limitations but is not as simple to perform as it may seem at first glance and requires close monitoring of stroke volume.

Although these tests may predict the increase in cardiac output after fluid administration, they do not provide information about the risks associated with fluid administration. Indeed, a high CVP, whatever its cause, reflects a high risk with fluid administration, with a high likelihood of further increasing capillary leak and back pressure on extrathoracic organs.

## Question 10: Should we try to decrease CVP in some conditions?

As elevated CVP levels may be associated with organ dysfunction [4, 5], one should try to maintain CVP as low as possible. This approach has been used in patients with ARDS, leading to fewer days of mechanical ventilation [18]. Importantly, this approach was applied only after initial hemodynamic stabilization, after resolution of shock. Accordingly, it may be reasonable to try to decrease CVP in the stabilization and de-resuscitation phases [23] as long as tissue perfusion is preserved.

## Conclusions

CVP values provide important information about the cardiocirculatory status of the patient and should not be abandoned. Use of CVP to guide fluid resuscitation has many limitations, but we believe it is wiser to understand and take into account these limitations rather than to discard CVP completely.

## Abbreviations

ARDS: Acute respiratory distress syndrome
CVP: Central venous pressure
Pms: Mean systemic pressure
PPV: Pulse pressure variation
SVV: Stroke volume variation
